# Treatment of Light Chain Deposition Disease Using Bortezomib-Based Regimen Followed by Thalidomide-Based Regimen in a Saudi Male

**DOI:** 10.1155/2016/7485695

**Published:** 2016-12-19

**Authors:** Bappa Adamu, Mushabab Al-Ghamdi, Mustafa Ahmad, Khaled O. Alsaad

**Affiliations:** ^1^College of Medicine, University of Bisha, Bisha, Saudi Arabia; ^2^Department of Medicine, King Fahad Medical City, Riyadh, Saudi Arabia; ^3^Department of Pathology and Laboratory Medicine, King Abdulaziz Medical City, Riyadh, Saudi Arabia

## Abstract

Light chain deposition disease (LCDD) is a rare illness with, as yet, no clear evidence-based guidelines for its treatment. To the best of our knowledge, LCDD has not been previously reported from Saudi Arabia. We present in this report, a 38-year-old Saudi male who presented with clinical features suggestive of hypertensive nephropathy but kidney biopsy later revealed the diagnosis of LCDD. His serum creatinine at presentation was 297 *μ*mol/L which came down to 194 *μ*mol/L on treatment with Bortezomib, Cyclophosphamide and Dexamethasone. His 24-hour protein excretion at presentation was 6 g/L which also came down to less than 1 g/day. He was later placed on Cyclophosphamide, Thalidomide, and Dexamethasone regimen because of persistent high titres of serum free light chains. He went into remission with undetectable serum free light chains and remained so for three years at the time of writing this report. We conclude that LCDD, though rare, does occur in Saudi population. The treatment of LCDD is challenging but the use of Bortezomib, a proteosome inhibitor, is promising. However, suboptimal response may require further treatment with other therapeutic options such as chemotherapy with alkylating agents or high-dose Melphalan with autologous stem cell transplant.

## 1. Introduction

Light chain deposition disease (LCDD) is a rare disease characterized by deposition of immunoglobulin fragments in various organs [1]. Renal involvement is almost universal while heart, liver, and other tissues are occasionally involved [2]. There are no established evidence-based guidelines for the treatment of LCDD. Various treatment approaches have been reported with variable results such as chemotherapy with alkylating agents/steroids and high-dose Melphalan with autologous stem cell transplantations [3]. Recently, there are few reports of the use of Bortezomib for the treatment of LCDD [2, 4–6]. We present in this report, a Saudi male who presented with LCDD and was treated with Bortezomib-based regimen followed by Thalidomide-based regimen with clinical and laboratory remission.

## 2. Case History

A 38-year-old Saudi male presented to our emergency room with history of generalized throbbing headache and nausea for two weeks. He had history of intermittent shortness of breath on exposure to dust for about three months but no other systemic complaints.

He sought medical advice in a private clinic, where he was found to have high blood pressure and elevated creatinine and was thus referred to our center. He had no history of hypertension prior to presentation at the referring hospital and has no symptoms of cardiac failure.

On presentation, general physical examination was unremarkable. Axillary temperature was 37.3°C and oxygen saturation was 97% on room air. Cardiovascular examination revealed a PR of 107/min, BP of 200/145 mm/Hg, normal JVP, and heart sounds. Examination of the abdomen, respiratory, and nervous systems was unremarkable.

Urine dipstick revealed 3+ protein and moderate blood. Urine microscopy revealed few crenated red blood cells and granular casts and no growth on culture. Blood counts were normal apart from mild normocytic normochromic anemia with a hemoglobin of 10.5 g/dL. Serum urea was 18.3 mmoles/L and creatinine was 297 *μ*mol/L while other electrolytes were normal. Twenty-four-hour urine protein was 6 g/day. Serum albumin was 33 g/L. Serum calcium, phosphate, and parathyroid hormones were normal. He was negative for hepatitis B, hepatitis C, and HIV. Antinuclear and antineutrophil cytoplasmic antibodies were negative. Serum complement levels C3/C4 were normal.

Urine protein electrophoresis showed generalized proteinuria and no evidence of Bence-Jones protein. Serum protein electrophoresis showed no monoclonal band. Serum free light chain was high with a kappa to lambda ratio of 24. Bone marrow aspiration revealed normocellular marrow with plasma cells of 4%.

Renal ultrasound revealed mild increase in echogenicity and renal sizes measuring 12.3 cm in bipolar diameters on the right and 13.3 cm in the left. Doppler renal scan was normal. His ECG was normal. Echocardiogram revealed mild concentric LVH, ejection fraction 55–60%, grade II diastolic dysfunction, right ventricle hypertrophy, pulmonary artery systolic pressure of 55 mm/Hg, and no pericardial effusion or valvular lesions. The intraventricular and intra-arterial septa were normal.

He then had renal biopsy which was subjected to light microscopy, immunofluorescence, and electron microscopy.

**Figure 1:**
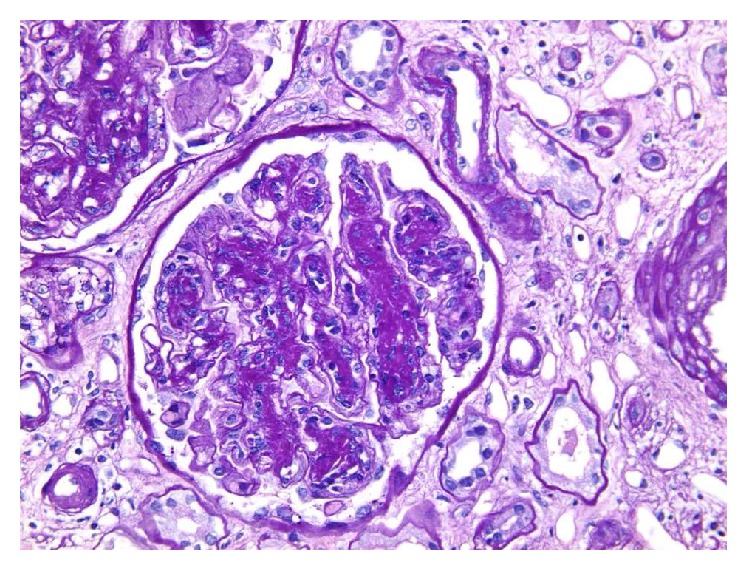
Photograph showing nodular accentuation of glomerular tufts and expanded mesangial areas (PAS ×200).

Light microscopic examination showed 26 glomeruli, of which 5 were globally sclerosed. The remaining glomeruli showed moderate to marked increase in mesangial matrix, moderate increase in mesangial cellularity, and marked nodular accentuation of the glomerular tufts (Figure 1).

The glomerular capillaries showed diffuse mild increase in thickness on PAS and Methenamine silver stains. No double contouring of the glomerular capillaries was seen. Capillary microaneurysmal formation was noted, along with focal segmental mild endocapillary hypercellularity. There was no evidence of glomerular necrotizing lesion, crescents formation, or microthrombi. Mild interstitial edema and lymphoplasmacytic inflammation were noted. The renal tubules showed diffuse mild to moderate increase in thickness of the tubular basement membranes (TBM). There was mild arterial sclerosis. There was no evidence of vasculitis. Cong Red special stain was negative for amyloidotic deposits.

Direct IF microscopy was reported as showing diffuse linear staining along the glomerular basement membranes (GBM) and TBM for Kappa light chain (3+). This was associated with mesangial staining of similar intensity. There was no staining for IgG, IgM, IgA, C3, C4, C1q, Lambda light chain, and albumin. Unfortunately, the IF slide was not preserved.

**Figure 2:**
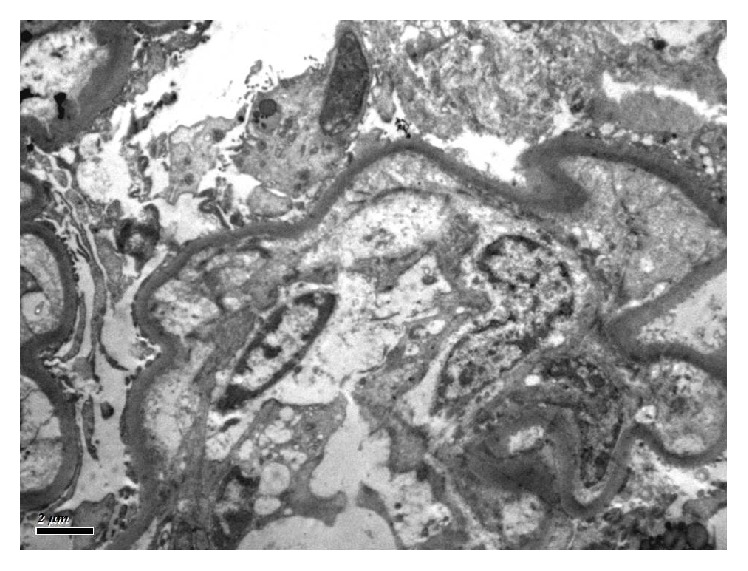
Transmission electron microscope photograph showing ultrastructural density along the GBM. (×10000).

Ultrastructurally, the increase in mesangial matrix was evident. The thickness of the GBM appeared within normal limits. Diffuse, continuous band of dense granules within the basement membranes, mainly in the inner aspect of the lamina densa was present (Figure 2). The same ultrastructural feature was also seen in the tubular basement membranes. The visceral epithelial cells showed effacement of the foot process over approximately 50% of the GBM.

The overall histologic features were consistent with light chain deposition disease of kappa type.

While on admission, the patient's serum creatinine rose from 297 to 331 *μ*mol/L over 3 days. He was started on methylprednisolone 500 mg daily for 3 days, followed by prednisolone 60 mg daily orally. Serum creatinine peaked at 382 over two days after pulse steroid and then declined to 194 at the time of discharge. He was later started on Bortezomib, Cyclophosphamide, and Dexamethasone regimen. He had Bortezomib 1.3 mg/M^2^ on days 1, 4, 8, and 11 on three weekly cycles, Cyclophosphamide 250 mg weekly, and Dexamethasone 40 mg weekly. He had six cycles of this regimen with steadily improving renal function and decline in proteinuria to 1.89 g/day. However, re-assessment showed that he still had high serum free light chains at 544 g/L with lambda of 23.4 and kappa to lambda ratio of 23.3. He was then started on four weekly cycles of Thalidomide, Cyclophosphamide, and Dexamethasone regimen. He had Thalidomide 100 mg daily, Cyclophosphamide 250 mg weekly, and Dexamethasone 40 mg weekly.

He had six cycles with complete remission as serum free light chains became undetectable and proteinuria remitted to less than 1 g/day. He has remained in remission for three years after treatment at the time of writing this report.

## 3. Discussion

Monoclonal immunoglobulin deposition disease (MIDD) is a rare disease characterized by the deposition of monoclonal immunoglobulin molecules in renal glomerular and tubular basement membranes. Three subtypes of MIDD are recognized depending on the composition of immunoglobulin deposits: LCDD, in which the deposits are composed of monoclonal light chains only; light and heavy chain deposition disease (LHCDD), in which the deposits are composed of monoclonal light and heavy chains; and heavy chain deposition disease (HCDD), in which the deposits are composed of monoclonal heavy chains only [3]. The patient presented in this report had LCDD variant of MIDD.

The first reports of MIDD in medical literature can be traced to Antonovych et al. [7] and Randall et al. [8] nearly 40 years ago. However, perhaps because of the rarity of MIDD, there are still no large prospective studies. We believe this case report represents the first report of LCDD from Saudi Arabia.

Clinically, most patients with LCDD (as illustrated in this report) present with nephrotic range proteinuria and rapidly deteriorating renal function [9].

Patients with LCDD may also have clinical features due to light chain deposition in other organ systems such as the nervous, endocrine, gastrointestinal, and cardiovascular systems [8]. The abnormal echocardiographic findings in this patient should raise the suspicion of cardiac involvement in LCDD. However, these abnormal findings could be from undetected hypertension as the patient presented with severe hypertension.

Renal histologic findings in LCDD on light microscopy are varied and not pathognomonic, including nodular glomerulosclerosis, glomerular and/or tubular basement membrane thickening, mesangial matrix increase, or even unremarkable glomeruli and tubules [1]. Membranoproliferative picture as seen in our patient has also been described [10]. Immunofluorescence microscopy in LCDD reveal linear deposits of monoclonal light chains on glomerular and tubular basement membranes as reported in this patient. Electron microscopy is essential for confirmation as light chain deposition can occur in other conditions apart from MIDD. Although there are no prospective trials to guide evidence-based treatment for LCDD, several treatment modalities have been tried for LCDD with varying outcomes. These treatment approaches range from conservative, expectant management to the use of chemotherapy and autologous stem cell transplant.

Recently, Bortezomib, a proteasome inhibitor, has been tried with varying response in patients with LCDD [2, 4–6].

The response in our index patient to Bortezomib-based chemotherapy was good from the renal point of view but he was placed on Thalidomide-based therapy because free light chain assay was still high after six cycles of Bortezomib-based regimen. The initial improvement in renal function despite persistent high serum free light chains could be as a result of partial response to treatment with reduction in proteinuria and control of blood pressure while the residual disease continues the secretion of free light chains. He achieved complete renal and hematologic remission after the Thalidomide-based therapy. He has remained in remission for three years at the time of writing this report.

## 4. Conclusion

We conclude that LCDD, though rare, does occur in Saudi population. The treatment of LCDD is challenging but the use of Bortezomib, a proteosome inhibitor, is promising. However, suboptimal response may require further treatment with other therapeutic options such as chemotherapy with alkylating agents or high-dose Melphalan with autologous stem cell transplant.
